# Force production and muscle activation during partial vs. full range of motion in Paralympic Powerlifting

**DOI:** 10.1371/journal.pone.0257810

**Published:** 2021-10-13

**Authors:** Tanise Pires Mendonça, Felipe José Aidar, Dihogo Gama Matos, Raphael Fabrício Souza, Anderson Carlos Marçal, Paulo Francisco Almeida-Neto, Breno Guilherme Cabral, Nuno Domingos Garrido, Henrique Pereira Neiva, Daniel Almeida Marinho, Mário Cardoso Marques, Victor Machado Reis

**Affiliations:** 1 Group of Studies and Research of Performance, Sport, Health and Paralympic Sports (GEPEPS), Federal University of Sergipe (UFS), São Cristovão, Sergipe, Brazil; 2 Department of Physical Education, Federal University of Rio Grande do Norte (UFRN), Natal, Brazil; 3 Research Center in Sports Sciences, Health Sciences and Human Development (CIDESD), University of Trás-os-Montes and Alto Douro, Vila Real, Portugal; 4 Research Center in Sports Sciences, Health Sciences and Human Development (CIDESD), University of Beira Interior, Covilhã, Portugal; Universidade Estadual Paulista Julio de Mesquita Filho - Campus de Bauru, BRAZIL

## Abstract

Paralympic Powerlifting is a sport in which the strength of the upper limbs is assessed through bench press performance in an adapted specific bench. It is therefore essential to optimize training methods to maximize this performance. The aim of the present study was to compare force production and muscle activation involved in partial vs. full range of motion (ROM) training in Paralympic Powerlifting. Twelve male athletes of elite national level in Paralympic Powerlifting participated in the study (28.60 ± 7.60 years of age, 71.80 ± 17.90 kg of body mass). The athletes performed five sets of 5RM (repetition maximum), either with 90% of 1RM in full ROM or with a load of 130% 1RM in partial ROM. All subjects underwent both exercise conditions in consecutive weeks. Order assignment in the first week was random and counterbalanced. Fatigue index (FI), Maximum Isometric Force (MIF), Time to MIF (Time) and rate of force development (RFD) were determined by a force sensor. Muscle thickness was obtained using ultrasound images. All measures were taken pre- and post-training. Additionally, electromyographic signal (EMG) was evaluated in the last set of each exercise condition. Post-exercise fatigue was higher with full ROM as well as loss of MIF. Full ROM also induced greater. EMG showed greater activation of the Clavicular portion and Sternal portion of pectoralis major muscle and lower in the anterior portion of deltoid muscle when full ROM was performed. Muscle thickness of the pectoralis major muscle increased post-exercise. We concluded that training with partial ROM enables higher workloads with lower loss of muscle function.

## Introduction

Paralympic Powerlifting (PP) is a paralympic sport that focus in maximum strength development and the best athletes are able to lift three times their body mass [1]. The paralympic bench press is a sport that is quite different from traditional weight lifting. Skilled athletes perform the movement with their lower limbs on the bench and may also use locked lashing belts [1].

As a strength modality, Paralympic Powerlifting training includes control of several variables, such as load, strength, movement duration and bar displacement velocity [2, 3]. Besides these critical variables, range of motion (ROM) can also be manipulated for strength gains [3, 4]. Studies which investigated the effects of performing partial or full ROM during strength training have shown that higher loads can be lifted while performing a bench press exercise with partial ROM [5–7]. Martinez-Cava et al., [8] suggested that lifting with full ROM enables higher strength gains when compared with lifting with partial or mixed ROM. In one of the earliest studies, Graves et al., [9] found that isometric strength gains in bilateral knee extension were similar throughout all knee angles when full ROM training occurred; whereas strength gains with partial ROM were limited to the joint angles involved in the partial ROM.

Weightlifters and others involved in resistance training commonly perform exercises with partial ROM to displace superior amounts of load or even to complete a target number of repetitions during a set. However, there is a lack in the literature regarding different ROM during weightlifting performed by Paralympic athletes.

Therefore, the aim of the present study was to compare the fatigue index, the maximum isometric force, the time to maximum isometric force, the rate of strength development, the muscle thickness and the activation of the muscles involved in partial vs. full range of motion (ROM) in Paralympic Powerlifting.

## Materials and methods

### Participants

The participants consisted of 12 male Paralympic Powerlifting athletes with 28.60 ± 7.60 years, 71.80 ± 17.90 kg and with a minimum training experience of 12 months. To enable seated weighing, weigh-in was performed on a digital electronic platform-type Michetti (Michetti, Brazil) with a maximum supported weight of 3000 kg and a size of 1.50 x 1.50m. All participants were national-level competitors who met the necessary prerequisites of the Brazilian Paralympic Committee to be eligible for the sport [1] and were ranked in the national top ten. The subjects´ mean best performance in bench press were 117.40 ± 23.37 kg, which corresponds to 1.67±0.28 times their body mass. Values above 1.4 are considered to define elite athletes, according to Ball and Wedman [10]. Five athletes presented spinal cord injury due to accidents with injuries below the eighth thoracic vertebra, four presented sequelae due to polio and three presented malformation in the lower limbs. Athletes were not included in the study if they i) reported the consumption of illicit substances (e.g., anabolic steroids), ii) presented a cardiac or metabolic disease, or iii) were involved in any process of rapid weight loss at the moment of recruitment. The athletes voluntarily participated in the study and signed an informed consent form in accordance with Resolution 466/2012 of the National Commission for Research Ethics (CONEP) of the National Health Council, and the ethical principles of the latest version of the Declaration of Helsinki (and the World Medical Association). The study was approved by the Ethics Committee of the Federal University of Sergipe (UFS) under protocol number 2.637.882.

### Experimental design

The study lasted for three weeks. In the first week the subjects were familiarized with measurement of electromyography, ultrasound and mechanical strength assessment procedures; and were also submitted to 1RM testing and re-testing. An interval of 48-h was kept between each session of testing on week 1.

**Fig 1:**
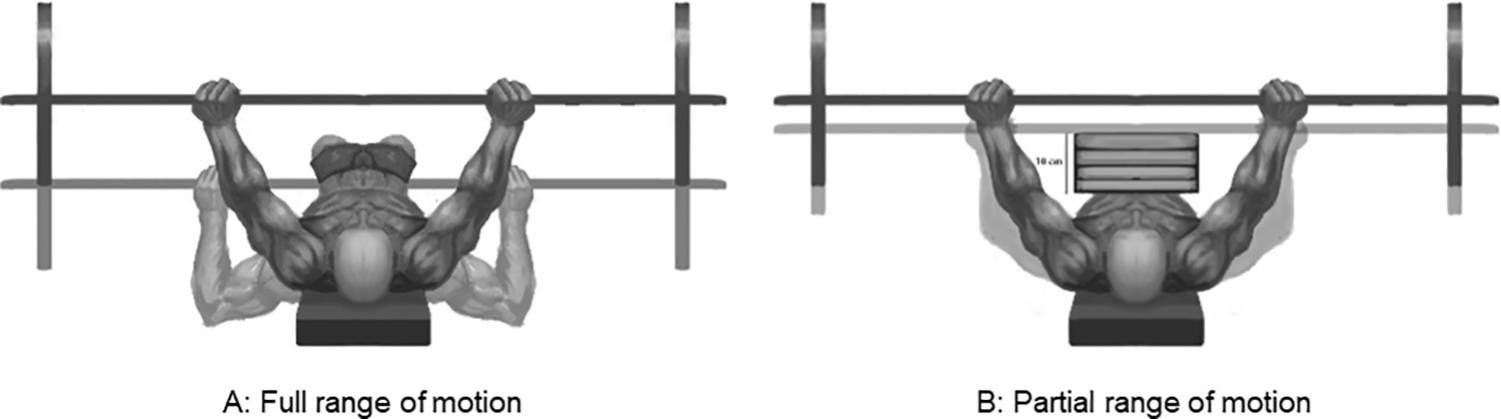
Bench press execution conditions. Partial ROM limited by four 2,5 cm thick boards.

On weeks 2 and 3 the individuals were submitted to two different exercise conditions in a counter balanced design. So, half of the athletes underwent a training session with full ROM and the other half underwent training with partial ROM. For the partial ROM, four wood boards 2.5 cm thick were used, as shown in Fig 1. For the bench press exercise, a standard adapted bench and an official Olympic bar (Eleiko, Sweden) approved by the International Paralympic Committee were used [1]. All measurements indicated above were performed prior and post- the training session. Subjects rested for one week and in week 3 they subjects underwent the experiment in a different condition of exercise. Table 1 displays the experimental design of the study.

**Table 1:** Experimental design.

**Week 1**	Session 1	Session 2	Session 3
	Familiarization	1RM	1RM re-test
**Week 2**	**Pre-Testing**	**Intervention**	**Post-Testing**
	Force measures Ultrasound image	Full or Partial ROM EMG in last set	Force measures Ultrasound image
**Week 3**	**Pre-Testing**	**Intervention**	**Post-Testing**
	Force measures Ultrasound image	Full or Partial ROM EMG in last set	Force measures Ultrasound image

ROM = range of motion; EMG = electromyography.

### Warm-up

Before each intervention, the athletes performed a 10.min warm-up for the upper limbs comprising three sets of 10 to 20RM in three exercises (shoulder abduction with dumbbells, elbow extension in the pulley, shoulder rotation with dumbbells) [11, 12]. Then a specific warm-up was performed on the bench with 30% of the individual´s 1RM load, comprising 10 slow repetitions (3-s eccentric + 1-s concentric) and 10 fast repetitions (1-s eccentric + 1-s concentric).

### 1-RM assessment

The 1RM test was performed in the week of familiarization (week 1), where each subject chose the load they believed could be lifted using the maximum effort. Weight increments were then added until the maximum load was reached. If the subject could not perform a single repetition, 2.5% of the load used in the test was subtracted [13]. The subjects rested between 3–5 minutes between attempts. The 1RM re-test was performed on a separate day (48-h later) and the highest record was taken as the 1RM.

### Partial range of motion exercise and testing

The subjects were submitted to the tests of FI, MIF, Time, RFD, thickness and muscular activation. Following this, after 10 minutes of rest [12], the athletes underwent an intervention in which they performed the five sets of 5RM, with a load of 130% 1RM with four boards (10 cm). After the test, the subjects rested for 10 minutes and repeated the tests of FI, MIF, Time, RFD, thickness and muscle activation. During the test, athletes were verbally encouraged to attain the target 5 repetitions per set [11]. Every subject was required to perform 5 sets and the load was not changed if they failed to complete the 5 repetitions.

### Full range of motion exercise and testing

The subjects were submitted to the tests of FI, MIF, Time, RFD, thickness and muscular activation. After 10 minutes of rest [12], they underwent an intervention of five sets of five maximum repetitions (5RM) with a load of 90% 1RM. After training, the subjects rested 10 minutes and the tests of PT, FI, Time, RFD, thickness and muscle activation were redone. During the test, athletes were verbally encouraged to attain the target 5 repetitions per set [11]. Every subject was required to perform 5 sets and the load was not changed if they failed to complete the 5 repetitions.

### Force measurement

**Fig 2:**
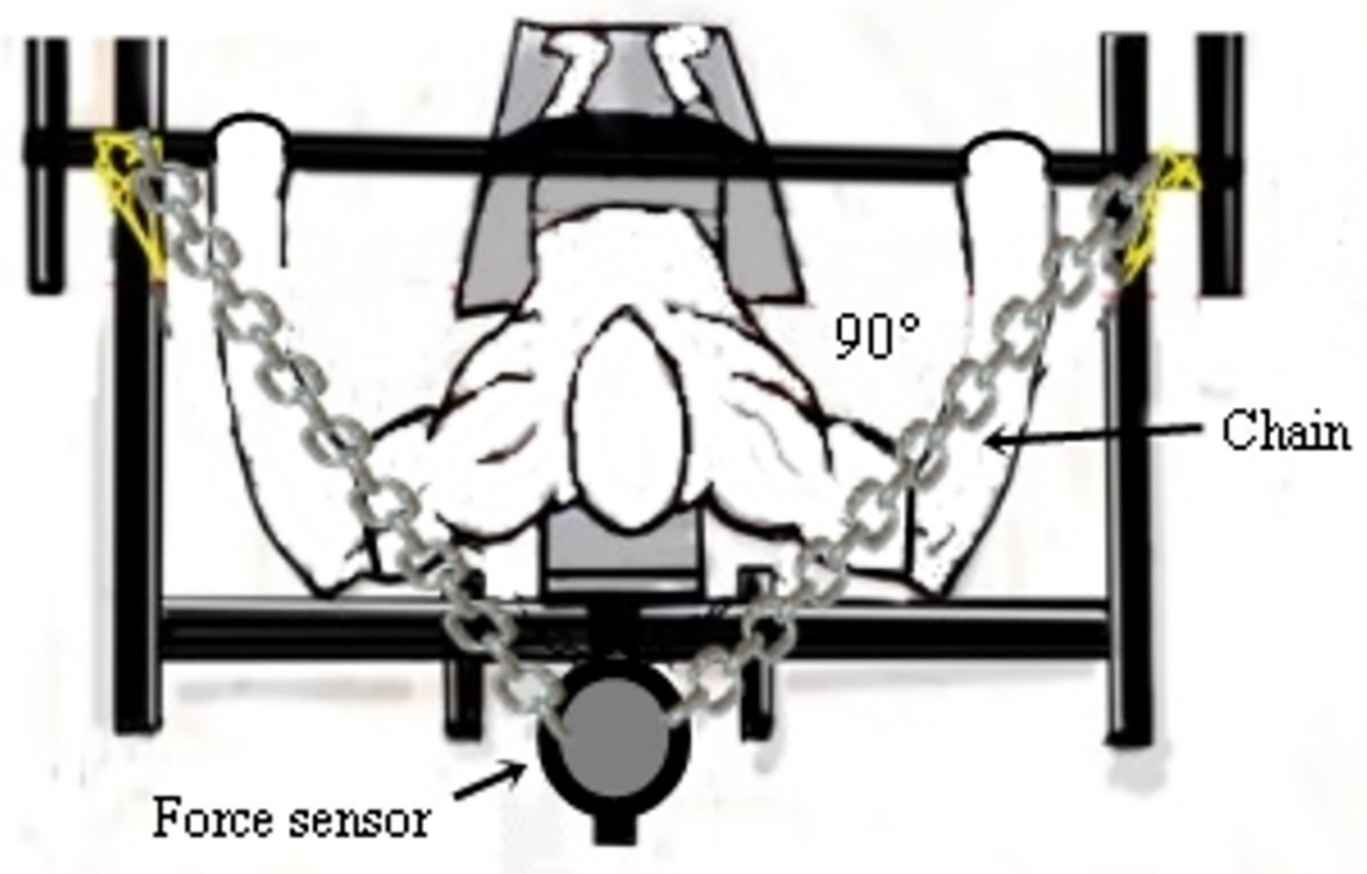
Placement of the force sensor.

Measurement of muscle strength, fatigue index (FI), Maximum Isometric Force (MIF), Time to MIF (Time) and Rate of Force Development (RFD) were determined by a force sensor PFMA 3010 (MuscleLab System, Langesund, Norway), fixed to the adapted bench press bench using Spider HMS Simond carabiners (Chamonix, France) with a rupture load of 21 KN. A steel chain with a breaking load of 2,300 kg was used to secure the force sensor to the seat (Fig 2). Perpendicular distance between force sensor and joint center was determined and used to calculate joint torques and fatigue index [14].

Maximum Isometric Force was measured by the maximum force generated by the upper limb muscles. The MIF was determined by the isometric force, measured by the force sensor and the adapted bench press bench, which was adjusted so that there was an elbow angle close to 90°, and at a distance of 15 cm from the starting point (chest to bar). Angle of the elbows was verified with a goniometer FL6010 (Sanny, Brasil). Participants were instructed to perform a single maximum movement seeking elbow extension (as soon as possible) and, after movement, to relax for MIF evaluation. For the fatigue index (FI) assessment, the same exercise was performed and the subjects were asked to maintain the maximum contraction for 10-s. FI was determined by FI = ((MIF final/MIF initial)-1,0)X100). The rate of force development (RFD) was measured through the relationship between time and force development (RFD = Δforce/Δtime), adapted from the adapted methodology of Milner-Brown, Mellenthin and Miller [15].

### Muscle thickness measurement

Muscle thickness (MT) was obtained using an Aloka^®^ SSD 500V ultrasound (US) with a 7.5 MHz electronic linear transducer (UST-5512U-7.5, 38 mm, Aloka^®^ (Tokyo, Japan). Ultrasound images were captured using a 90 dB gain and a magnification that allows a depth of 42.0 mm of the Pectoralis major muscle. Images were acquired between the Sternal and Clavicular portion. Participants remained relaxed for 10 min to allow fluid distribution before and after training [16]. Participants were asked to relax the upper limbs and thorax muscles as much as possible throughout the procedures and the lower limbs were fixed with Velcro to avoid interference of possible muscle spasms. The uniformity of the compressions was standardized real-time by examination of the device screen [17, 18]. The tests were performed on the dominant side [19, 20].

### Muscle activation measurement

Maximum isometric force measurement and electromyographic signal were evaluated simultaneously. Surface electromyography (EMG) is a noninvasive technique with no contraindications, which assesses the action potential of muscle fiber activity over which the sensors are placed [21].

EMG analysis used EMG System EMG 432C muscle equipment. EMG targeted the Pectoralis major sternal portion (LSP) and clavicular portion (CP), the anterior deltoid (AD), and the long head triceps brachii (LHTB), which were captured during maximal voluntary 10-s isometric contractions (MVIC). There was an elbow angulation of approximately 90°, with a distance of 15.0 cm from the bar to the stern [14]. The surface electrodes (double, bipolar and disposable) were positioned at an average distance between the motor point and the tendon of the evaluated muscles, parallel to the muscle fibers with a distance of 20 mm between them. A reference electrode fixed to the olecranon was also used, according to SENIAM recommendations (Surface Electro Myo Graphy for the Non-Invasive Assessment of Muscles) [19]. Prior to testing, asepsis and local trichotomy were performed and then the measurement points were marked with a felt-tip pen. For signal rectification, the high-pass and low-pass filters (500–10) and the offset were used. The largest signal of the (MVIC) and the mean square root mean were used for signal presentation, and the signal normalization was made from the maximum. EMG was evaluated in the last set of each of the training interventions (full and partial ROM).

### Statistical analysis

The statistical analysis was performed using the Statistical Package for Social Science, version 22.0. Normality and sphericity assumptions were confirmed with Shapiro Wilk and Mauchly tests, respectively and were then followed by a 2x2 repeated measures ANOVA, which was performed on the following dependent variables: Rate of force development, maximum isometric force, fatigue index, time to maximum isometric force and muscle thickness in clavicular and sternal portions of pectoralis major. The factors (interaction terms) were the exercise condition (full vs. partial ROM) and the moments in time of assessment (pre- vs. post-training). In the 2x2 ANOVA, the effect size was given by the η_p_^2.^ When significant interactions were identified, post-hoc pairwise analysis was performed along with Cohen´s d for effect size. EMG records post-training with the exercise conditions were compared with paired t-test. The level of statistical significance was set at p ≤ 0.05. Results are presented as means (X) ± standard deviation (SD).

## Results

Table 2 presents descriptive for every variable in the various moments of time and exercise condition. 2x2 ANOVA exhibit a significant effect of moment in time in rate of force development with decreased values post-exercise (F = 28.216; p<0.001; η_p_^2^ = 0,720). The same was observed for time to maximum isometric force (F = 8.234; p = 0.014; η_p_^2^ = 0.437).

**Table 2:** Rate of Force Development (RFD), Maximum Isometric Force (MIF), Fatigue Index (FI), Time to maximum isometric force (Time) (sec) and muscle thickness in clavicular and sternal portions of pectoralis major in the different exercise conditions.

	Partial ROM pre	Partial ROM post	Full ROM pre	Full ROM post
	mean ± SD	mean ± SD	mean ± SD	mean ± SD
	(CI 95%)	(CI 95%)	(CI 95%)	(CI 95%)
**RFD (N.m.s** ^**-1**^ **)**	1129.42±354.72	640.32±360.08	1238.22±196.70	602.13±261.75
	(903.04–1353.80)	(411.54–869.10)	(1113.24–1363.20)	(435.82–768.44)
**MIF**	1154.19±303.00	1119.84±253.293	1244.31±434.72	771.79±197.54
**(N)**	(961.67–1346.71)	(958.91–1280.77)	(968.10–1520.52)	(646.28–897.31)
**FI**	66.77±8.35	76.61±4.44	66.75±10.60	61.06±14.02
**(%)**	(61.46–72.08)	(73.79–79.43)	(60.01–73.48)	(52.15–69.97)
**Time**	0.72±0.22	1.01±0.35	0.64±0.17	1.03±0.62
**(sec)**	(0.58–0.87)	(0.79–1.23)	(0.54–0.75)	(0.63–1.43)
**Clavicular (cm)**	2.24±0.42	3.03±0.48	2.09±0.49	3.34±0.52
	(1.97–2.50)	(2.73–3.33)	(1.78–2.40)	(3.01–3.67)
**Sternal (cm)**	2.15±0.45	2.90±0.40	2.41±0.54	3.13±0.59
	(2.25–2.82)	(2.64–3.15)	(2.07–2.75)	(2.75–3.51)

Values are means ± standard deviations (SD), with confidence intervals (CI) in parentheses.

ROM = range of movement; CI = Confidence interval.

In the remaining variables significant interaction of exercise condition vs moment in time were detected and therefore pairwise comparisons between moments in time were investigated for each exercise conditions separately. Maximal isometric force decreased in the full Rom condition (p<0.001; d = 3.53), and also, though with a smaller effect, in partial ROM (p<0.001; d = 1.85). The fatigue index increased significantly solely in the partial Rom condition (p<0.001; d = 1.65), but did not in full ROM (p = 0.079; d = 0.59). The clavicular portion of the pectoralis major muscle thickness increased from pre- to post in both exercise conditions, with greater effect in full ROM (p<0.001; d = 3.33), compared with partial ROM (p<0.001; d = 2.34). Finally, the sternal portion of the pectoralis major muscle thickness also increased from pre- to post in full ROM (p<0.001; d = 1.71) and in partial ROM p<0.001; d = 2.36).

**Fig 3:**
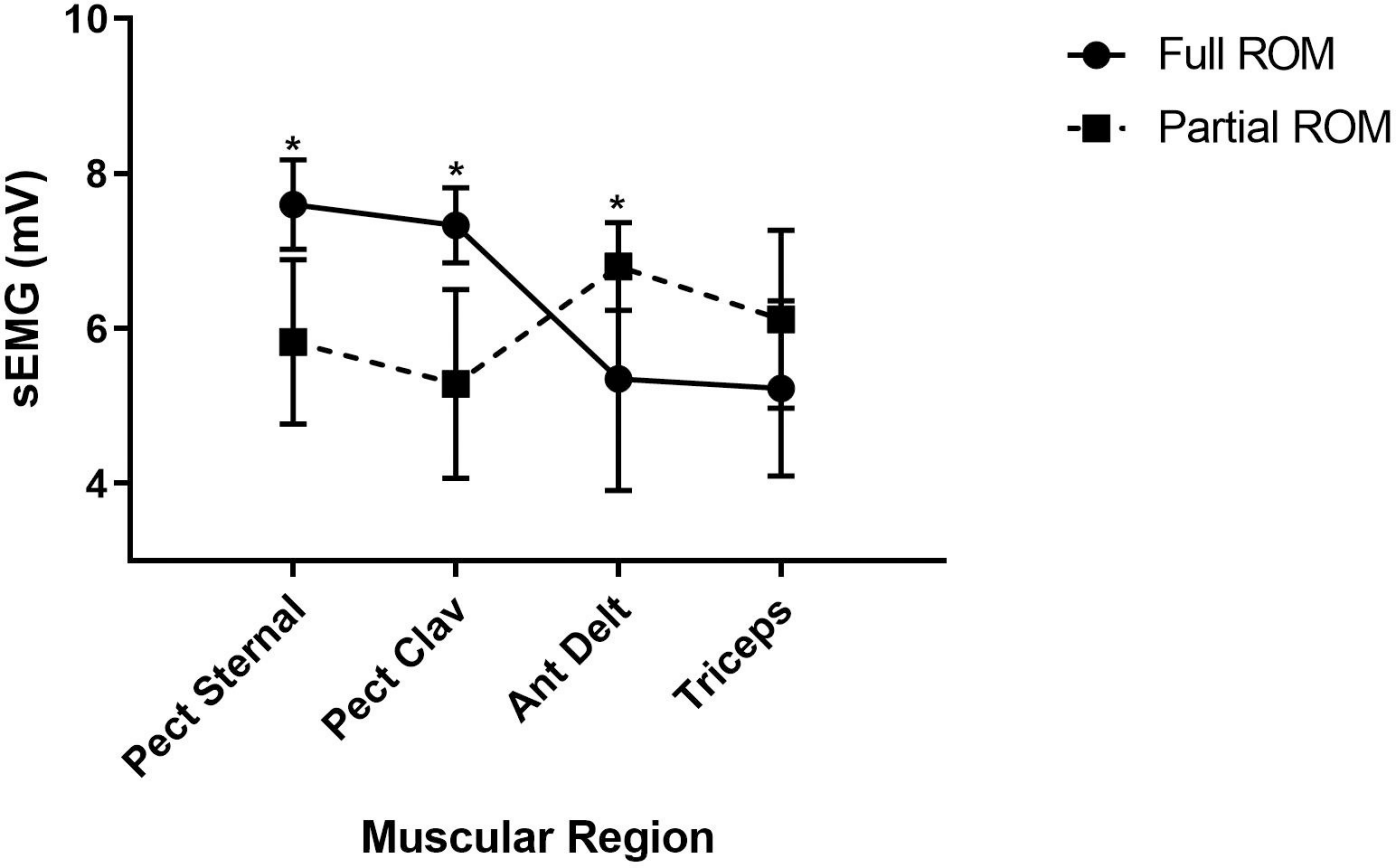
Comparisons of electromyographic signal. sEMG = Electromyographic signal; Ant Delt: anterior portion of the deltoid muscle; Pect Clav: clavicular portion of the pectoralis major muscle; Pect Sternal: sternal portion of the pectoralis major muscle; * p <0.05.

Fig 3 shows the values of electromyography (sEMG) performed in the last set of each exercise condition. When full ROM training was performed, muscle activation was higher in both portions of the pectoralis major muscle, compared with partial ROM. Contrarily, in triceps muscle, it was higher when training was performed with partial ROM, compared with full ROM. Activation among different muscles varied less training with partial ROM, though it was higher in the anterior portion of the deltoid when compared with the clavicular portion of the pectoralis major muscle.

## Discussion

The aim of the present study was to compare the fatigue index, the maximum isometric force, the time to maximum isometric force, the rate of strength development, the muscle thickness during training with partial vs. full range of motion (ROM) in Paralympic Powerlifting.

The main results of the present study were that training with full ROM induced greater muscle signs of fatigue (greater decrease in maximum isometric force and greater increase in fatigue index).

### Force measurements

Training with full ROM seems to induce a greater muscle fatigue, as shown in the present study. Moreover, training with partial ROM enables higher training loads. Indeed, Sullivan et al., [22], have observed greater torques during training with partial ROM. Clark et al., [23] also found that the maximum force produced in the bench press, through the 6RM test, was greater when smaller ranges of motion were performed. These also demonstrated that it is possible to achieve a better performance using partial ROM in a 1RM test, when compared with the full ROM [23, 24]. They concluded that resistance training programs that emphasize a high force generation in different regions of the ROM can provide benefits to the performance of athletes, as well as help in control of movement. Our results are consistent with this rationale. Furthermore, it was also necessary to investigate whether this larger force generated during partial ROM would results in changes in muscle thickness.

### Muscle thickness

Regarding the edema measured through ultrasonography, it was found that the muscle thickness changed significantly from pre- to post-test exercise in both training conditions. Changes were greater when the subjects performed the full ROM training (especially in the clavicular portion of deltoid muscle) and a significant interaction between time of measurement and exercise conditions was evident. Indeed, post-exercise muscle thickness was larger in the full ROM condition, despite a lower load being lifted.

Our study partially corroborates that by Pinto et al., [25], who used elbow flexion exercise with partial and full ROM to assess changes in strength and muscle thickness of the elbow flexors of the right limb for 10 weeks. The authors observed that the full ROM generated more hypertrophy compared to the partial repetition. As in our study, the thickness values were also larger in full ROM. In addition, the aforementioned authors [25], analyzed the biceps curl exercise with partial and full ROM after 10 weeks of training, and full ranges of motion proved to be more prone to induce post-exercise muscle edema.

The fact that the subjects were able to move a higher load (130% 1-RM) in partial ROM with a smaller muscle edema, seems to favor this training strategy when maximum strength is the main goal. Indeed, the body of knowledge on recovery mechanisms post-inflammation supports the rationale that larger edema requires enlarged recovery time [26].

### Muscle activation

Previous studies on bench press exercise [27] and on elbow flexion exercise [25] showed that strength gains were larger when partial ROM was involved. Although these have not measured muscle activation with EMG, their results could indicate that partial ROM could enhance larger benefits due to a higher activation of motor units. Da Silva et al., [28] also used EMG to assess muscle activation at different angles in the squat exercise. The gluteus maximus, biceps femoris and soleus muscles had a greater activation in the partial movement when compared with full ROM.

In the present study, muscle activation was different between the two exercise conditions. During full ROM pectoralis major muscle was the most active muscle group, whereas in the partial ROM it was triceps and deltoid muscles. On the other hand, Lander et al., [29] found that the point of failure in the bench press exercise occurs during the concentric phase and that the range of movement where it occurs does not vary much regardless of the intensity (75 or 90% of 1 RM). Moreover, Wilson et al., [30] confirmed that the concentric phase of the bench press is especially difficult due to mechanical disadvantage. Altogether, these studies, seem to indicate that training with full ROM may impair extremely high neuromuscular recruitment, which is usually warranted in powerlifting.

The main limitation of the present study was the small number of participants. The specificity of this populations does not enable large scale studies and therefore we must rely on the accumulation of more contributions before a clear tendency is established. Another limitation is that the differences herein do not necessarily support possible differences in adaptations to partial ROM training as apposite to full ROM training.

## Conclusion

Training through partial range of motion has been used in Paralympic Powerlifting. However, to the present, possible differences and / or similarities between this method and that with full range of motion were not clear. Post-exercise fatigue indicators were higher with full ROM (as given by a greater fatigue index and by a greater loss of maximal isometric force). We conclude that training with partial ROM enables handling of higher workloads with lower loss of muscle function.

## Supporting information

S1 Dataset (XLSX)
